# Unusual exanthema combined with cerebral vasculitis in pneumococcal meningitis: a case report

**DOI:** 10.1186/1752-1947-5-410

**Published:** 2011-08-24

**Authors:** Theonimfi Tavladaki, Anna-Maria Spanaki, Stavroula Ilia, Elisabeth Geromarkaki, Maria Raissaki, George Briassoulis

**Affiliations:** 1 Paediatric Intensive Care Unit, University Hospital of Heraklion, University of Crete, Heraklion, Crete, Greece; 2 Department of Radiology, University Hospital of Heraklion, University of Crete, Heraklion, Crete, Greece

## Abstract

**Introduction:**

Bacterial meningitis is a complex, rapidly progressive disease in which neurological injury is caused in part by the causative organism and in part by the host's own inflammatory responses.

**Case presentation:**

We present the case of a two-year-old Greek girl with pneumococcal meningitis and an atypical curvilinear-like skin eruption, chronologically associated with cerebral vasculitis. A diffusion-weighted MRI scan showed lesions with restricted diffusion, reflecting local areas of immunologically mediated necrotizing vasculitis.

**Conclusions:**

Atypical presentations of bacterial meningitis may occur, and they can be accompanied by serious unexpected complications.

## Introduction

Neurological injury in *Streptococcus pneumoniae *meningitis can be due to meningeal inflammation, cerebral edema, necrosis and intracranial hemorrhage. There is a widely held belief that cerebral infarction after bacterial meningitis is always caused by vasculitis; however, evidence for this is weak. Vergouwen *et al*. hypothesized that diffuse cerebral intravascular coagulation is an additional explanation for cerebral infarction in patients with pneumococcal meningitis [1]. At the molecular level, *S. pneumoniae *cell walls have been shown to induce cerebrovascular endothelial cells, microglia, and meningeal inflammatory cells to release cytokines, chemokines and reactive oxygen species [2]. These include tumor necrosis factor α, interleukins 1 and 6, platelet-activating factor, peroxynitrites, matrix metalloproteinases and urokinase plasminogen activator. Release of such bioproducts is believed to play a role in the development of disseminated intravascular coagulation in the setting of pneumococcal sepsis. To the best of our knowledge, we present a previously-unreported association of an exaggerated host response, as shown by the development of vasculitis, with an unusual rash in a child with pneumococcal meningitis.

## Case presentation

**Figure 1:**
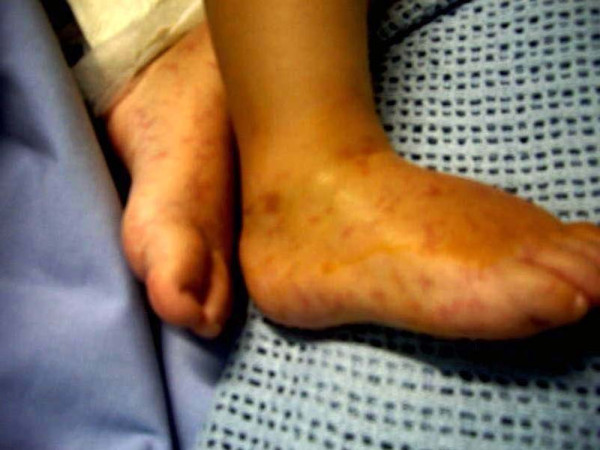
**Multiple non-hemorrhagic erythematous flat macules on the dorsal and plantar aspects of feet**.

**Figure 2:**
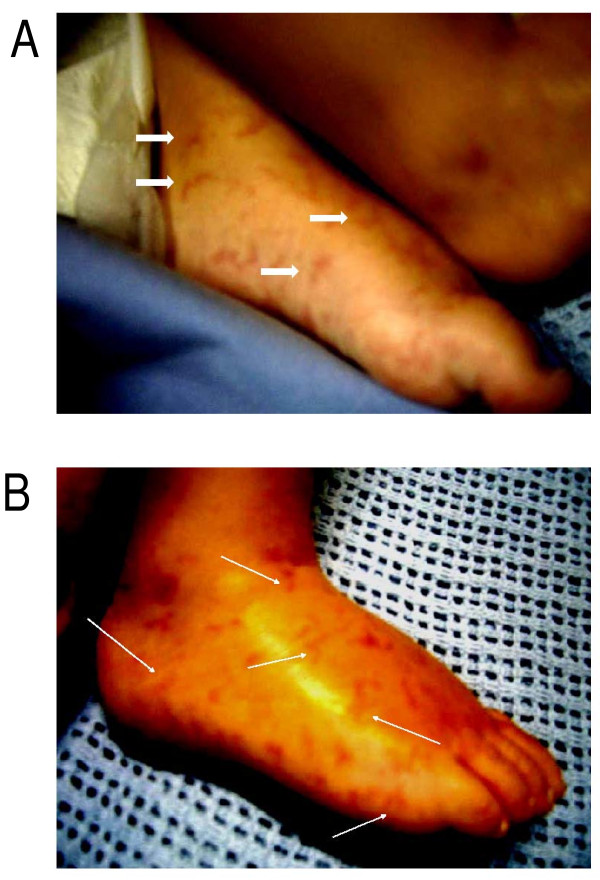
**Confluent elongated skin lesions (A, arrows) with curvilinear projections (B, arrows) at the time of isolation of *Streptococcus pneumoniae *in blood and cerebrospinal fluid**.

A two-year-old Greek girl was referred to our Pediatric Intensive Care Unit (PICU) with a two-day history of fever (39.3°C), vomiting, reduced appetite for feeding and seizures. A physical examination showed nuchal rigidity, a decreased level of consciousness and multiple erythematous, flat macules present on her hands and the dorsal and plantar aspects of her feet (Figure 1), taking a curvilinear appearance (Figure 2A, B). Our patient had an unremarkable medical history; she had not been vaccinated for *S. pneumoniae*.

**Figure 3:**
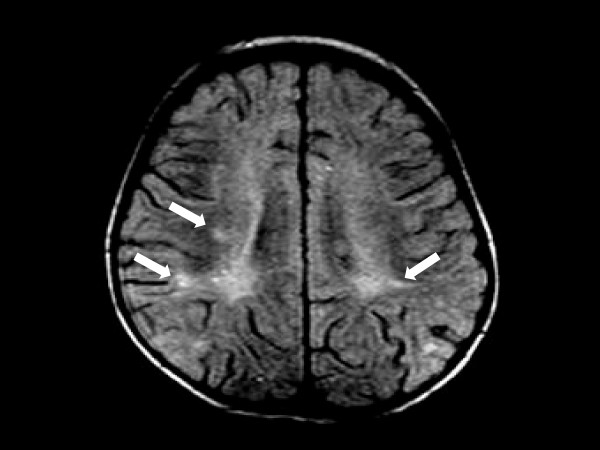
**MRI scan showing ill-defined hyperintense lesions at the peri-ventricular and subcortical white matter (arrows) that were identified shortly after the skin eruption and the *Streptococcus pneumoniae *growth**.

A complete blood cell count revealed 18,000 cells/μL white blood cells (neutrophils 80%, leukocytes 17%), her C-reactive protein serum level was 28.87 mg/dL, and pronounced coagulation disturbances were detected (prothrombin time: 15.4 seconds; activated partial thromboplastin time: 33 seconds; international normalized ratio: 1.38; fibrinogen: 375 mg/dL, D-dimers: 91.63 mg/dL). Results of a lumbar puncture showed white blood cells at 40 cells/mm^3^, a total protein content of 169 mg/dL and hypoglycorrhachia of 2 mg/dL. Gram-staining results revealed the presence of Gram-positive cocci in pairs. Two days after admission, blood and cerebrospinal fluid cultures yielded pure growth of vancomycin susceptible (MIC # 1 μg/mL, 25 mm) and penicillin susceptible (MIC # 0.12 μg/mL) *Streptococcus pneumoniae*. Serotype 23F was identified by PCR from two blood samples and in the first sample of cerebrospinal fluid (CSF). The same isolate was also cultured from our patient's throat. IgG subclasses were normal and the results of an HIV test were negative. Due to the lack of clinical improvement, an urgent diffusion-weighted MRI scan was performed six days after admission. The MRI showed ill-defined hyperintense lesions at the peri-ventricular and white matter, exhibiting restricted diffusion (Figure 3).

Boluses of intravenous fluids, fresh frozen plasma and intravenous dexamethasone (0.15 mg/kg) were given, immediately followed by systematic administration of ceftriaxone (100 mg/kg/day) and vancomycin (60 mg/kg/day). Due to persistent drowsiness and further clinical deterioration, a second lumbar puncture was taken. The results of this were 90 leukocytes/mm^3^, a glucose level of 36 mg/dL, and protein 124 mg/dL, whereas a further CSF culture did not reveal any isolation. Aiming at better permeability through the blood brain barrier, intravenous rifampicin (40 mg/kg/day, MIC # 1 μg/mL, 27 mm) was added. Although the responsible isolate was sensitive to the antibiotics administered, our patient showed a slow clinical response; consequently the combined antibiotic regimen was administered for a total of 14 days after therapy initiation. Her fever and atypical rash started resolving after the first week. Our patient made a full neurological recovery, apart from bilateral hearing impairment confirmed by brain stem response.

## Discussion

Following usage of the pneumococcal conjugate vaccine in children, the incidence of invasive pneumococcal disease (IPD) has declined in both children and adults (reflecting herd immunity). Since our patient's responsible serotype is included in all types of current *S. pneumoniae *vaccines, her life-threatening atypical bacterial infection could have been avoided if the child had been appropriately vaccinated. (Following the introduction of heptavalent pneumococcal conjugate vaccine (PCV7), the incidence rates of IPD caused by vaccine serotypes declined across all age groups [3,4].)

Although atypical presentations of bacterial meningitis still occur, emergency or community physicians are rarely involved [5]. Only an atypical exanthema (erythema nodosum) associated with meningitis (due to *Chlamydia pneumonia*) has been reported in the literature [6]; to the best of our knowledge such an unusual exanthema, presented in clusters of curvilinear skin lesions and associated with severe pneumococcal infection, has never been described previously. Absence of hemorrhagic rash has been recently reported as one of the most significant clinical predictors of childhood pneumococcal meningitis [7]. Regardless, such an atypical skin eruption, chronologically associated with cerebral vasculitis, has not been described in a child with pneumococcal meningitis to date. However, a low CSF glucose level, which was profoundly low (2 mg/dL) in our patient, is an established significant risk factor for hearing loss after pneumococcal meningitis [8,9].

As in our patient, in adult patients with meningoencephalitis caused by *S. pneumoniae*, diffusion-weighted MRI may show lesions with restricted diffusion, reflecting local areas of ischemia with cytotoxic edema secondary to immunologically mediated necrotizing vasculitis and thrombosis [10]. Conventional angiography and magnetic resonance angiography may show tapering and stenosis of arteries [11]. Importantly, in a series in adults, pneumococcal meningitis-associated arterial (21.8%) or venous (9.2%) cerebrovascular complications have been shown to develop more frequently than previously reported [12]. Other reported findings from the same study were subarachnoid hemorrhages in association with angiographic evidence of vasculitis (9.2%) and acute spinal cord dysfunction due to myelitis (2.3%). Delayed cerebral thrombosis has also been described in adult patients with pneumococcal meningitis, with pathology suggesting an immunological reaction targeting cerebral blood vessels [13].

*S. pneumoniae *bacteria do not readily penetrate the pia and invade the brain. However, the interaction between *S. pneumoniae *and the host results in meningeal inflammation, vascular injury, disruption of the blood-brain barrier, vasogenic, interstitial and cytotoxic edema, and disruption of normal CSF flow. Many of the neurological and systemic conditions that contribute to morbidity and mortality in pneumococcal meningitis, in particular vascular injury and cerebral edema, have already been set in motion by the time antibiotics are administered. So even if antibiotic treatment is started early and the bacteria are drug sensitive, as in our patient's case, unfavorable outcomes and severe neurological sequelae of bacterial meningitis frequently still occur. Treatment options to suppress the inflammatory cascade causing neuronal injury include corticosteroids, as they exert various immunomodulatory actions. Although previously controversial, as steroids reduce antibiotic penetration into the CSF, meta-analysis of trial data now support treatment with a short course of adjunctive therapy with the corticosteroid dexamethasone to improve outcome and partially prevent neurological sequelae from bacterial meningitis in adults and children [14]; this is however only achieved when given early in the disease course and when started with or before parenteral antibiotics [14]. It has been recently suggested that dexamethasone inhibits increase of CSF soluble tumor necrosis factor 1 levels after antibiotic therapy in bacterial meningitis, an important indicator of neurological sequelae in bacterial meningitis [15].

## Conclusions

The interaction between *S. pneumoniae *bacteria and the host results not only in meningeal inflammation but also in vascular injury. Early administration of dexamethasone and empiric antibiotic treatment should begin in all cases to prevent neurological sequel and hearing loss associated with low CSF glucose levels. Accordingly, the presence of an atypical rash should not deter the physician from a clinical suspicion of this potentially fatal pneumococcal infection. Brain MRI scans and/or angiography, as well as CSF findings in conjunction with the clinical course of this life-threatening disease, may dictate appropriate treatment adjustments. Importantly, to the best of our knowledge, an atypical skin eruption chronologically associated with cerebral vasculitis has not been described previously. However, with routine effective use of pneumococcal conjugate vaccines a general decline in IPD, antibiotic non-susceptibility and vaccine serotypes was observed.

## Consent

Written informed consent was obtained from the patient's legal guardian for publication of this case report and any accompanying images. A copy of the written consent is available for review by the Editor-in-Chief of this journal.

## Competing interests

The authors declare that they have no competing interests.

## Authors' contributions

GB, TT, SI, EG, and AMS were responsible for the management of our patient; MR performed the MRI, and interpreted and discussed findings; GB, TT and AMS participated in the study design and coordination and helped draft the manuscript. All authors read and approved the final manuscript.
